# Enhanced visible light photocatalytic activity of Fe_2_O_3_ modified TiO_2_ prepared by atomic layer deposition

**DOI:** 10.1038/s41598-020-70352-z

**Published:** 2020-08-10

**Authors:** Yan-Qiang Cao, Tao-Qing Zi, Xi-Rui Zhao, Chang Liu, Qiang Ren, Jia-Bin Fang, Wei-Ming Li, Ai-Dong Li

**Affiliations:** 1 National Laboratory of Solid State Microstructures, Jiangsu Key Laboratory of Artificial Functional Materials, Materials Science and Engineering Department, College of Engineering and Applied Sciences, Collaborative Innovation Center of Advanced Microstructures, Jiangsu Key Laboratory of Artificial Functional Materials, Nanjing University, Nanjing, 210093 People’s Republic of China; 2 Institute of Micro-Nano Photonic and Beam Steering, School of Science, Nanjing University of Science and Technology, Nanjing, 210094 People’s Republic of China; 3 Jiangsu Leadmicro Nano-Technology Co., Ltd., Wuxi, Jiangsu People’s Republic of China

**Keywords:** Photocatalysis, Nanoparticles

## Abstract

In this work, commercial anatase TiO_2_ powders were modified using ultrathin Fe_2_O_3_ layer by atomic layer deposition (ALD). The ultrathin Fe_2_O_3_ coating having small bandgap of 2.20 eV can increase the visible light absorption of TiO_2_ supports, at the meantime, Fe_2_O_3_/TiO_2_ heterojunction can effectively improve the lifetime of photogenerated electron–hole pairs. Results of ALD Fe_2_O_3_ modified TiO_2_ catalyst, therefore, showed great visible light driven catalytic degradation of methyl orange compared to pristine TiO_2_. A 400 cycles of ALD Fe_2_O_3_ (~ 2.6 nm) coated TiO_2_ powders exhibit the highest degradation efficiency of 97.4% in 90 min, much higher than pristine TiO_2_ powders of only 12.5%. Moreover, an ultrathin ALD Al_2_O_3_ (~ 2 nm) was able to improve the stability of Fe_2_O_3_-TiO_2_ catalyst. These results demonstrate that ALD surface modification with ultrathin coating is an extremely powerful route for the applications in constructing efficient and stable photocatalysts.

## Introduction

A rapid industrial development driven by unsustainable technology advances can cause plenty of industrial sewage, spreading chemical hazards into water resources. As a result, water pollution has emerged as one of the most serious environmental issues worldwide^1–4^. Photocatalytic oxidation technology has shown great prospects in removing the toxic and harmful contaminants in aqueous environment^5–7^. Semiconductors (e.g. TiO_2_, ZnO, SnO_2_) have been widely researched for organic pollutant degradation, however, the large band gap hinders their practical applications^8–12^. For example, TiO_2_ with band gap of 3.2 eV can only absorb the ultra-violet light, accounting for only 4–5% of entire solar spectrum^13^. Therefore, various visible light sensitive photocatalysts has also been widely explored, such as g-C_3_N_4_, BiVO_4_, CdSe, Bi_2_WO_6_^14–19^. On the other hand, TiO_2_ is recognized as one of the excellent materials owning to its good inertness, eco-friendly, low cost, strong oxidizing power, and long-term stability against photo and chemical corrosion^9,13,20–22^. Thus, plenty of works have been made to extend the absorption spectrum of TiO_2_ to visible light so to make a full use of solar spectrum. Several different approaches can be employed, including doping^23–26^ and coupling with small band gap semiconductors or metals^27–30^.

Small band gap semiconductors not only increase the absorption of visible light but also inhibit photo-generated electrons-holes recombination when constructed as a semiconductor/semiconductor heterojunction structure, thus improving the photocatalytic performance dramatically^31^. Therefore, various TiO_2_ based heterojunction photocatalysts have been proposed for visible light photocatalysis, including NiO/TiO_2_^32,33^, Ag_2_O/TiO_2_^34^, CdTe/TiO_2_^35^, C_3_N_4_/TiO_2_^36^, Bi_2_O_3_/TiO_2_^37^, Cu_2_O/TiO_2_^38^, Fe_2_O_3_/TiO_2_^39^, etc. For Fe_2_O_3_/TiO_2_ heterojunction photocatalysts, a variety of composites have been investigated, such as Fe_2_O_3_ nanoparticles on TiO_2_ nanotube^40^, Fe_2_O_3_/TiO_2_ nanoparticles^41^, TiO_2_ coated cubic Fe_2_O_3_^42^, Fe_2_O_3_ nanosheet/TiO_2_ hollow sphere^39^, and Fe_2_O_3_ coated TiO_2_^43^. For instance, Lin et al. demonstrated that Fe_2_O_3_ coating can effectively enhance the visible light photocatalytic activity of TiO_2_^43^. Various fabrication methods were applied, including hydrothermal or solvothermal process and sol–gel, to prepare the heterojunction photocatalysts^44–46^. Nevertheless, precise control of the interface between Fe_2_O_3_ and TiO_2_ at atomic level by conventional methods remain challenges.

Atomic layer deposition (ALD) is a unique and promising thin film deposition technique based on self limited and saturated surface chemisorption reactions. It can deposit ultrathin, conformal, and uniform layers at sub-nanometer scale, which has attracted great attentions in surface engineering of nanostructures over the years^47–49^. In catalysts design, ALD enables a conformal layer with precise thickness control and tunable film composition onto another nanostructures with high aspect ratio^50^. The ALD coating can work as photo-active materials^51,52^ or surface protection layer^53,54^. Herein, we modified the commercial anatase TiO_2_ powders with ultrathin Fe_2_O_3_ surface coating by ALD. The photocatalytic performance was investigated by visible light degradation of methyl orange (MO). The ultrathin Fe_2_O_3_ coating can enhance the absorption of TiO_2_ supports for visible light. Fe_2_O_3_ modified TiO_2_ powders show much better visible light photocatalytic degradation of MO than pristine TiO_2_. A possible mechanism for improved photocatalytic performance is proposed. In addition, an ultrathin ALD Al_2_O_3_ (~ 2 nm) was used to promote the long-term durability of TiO_2_@Fe_2_O_3_ catalyst.

## Methods

### ALD deposition on TiO_2_ powders

**Figure 1:**
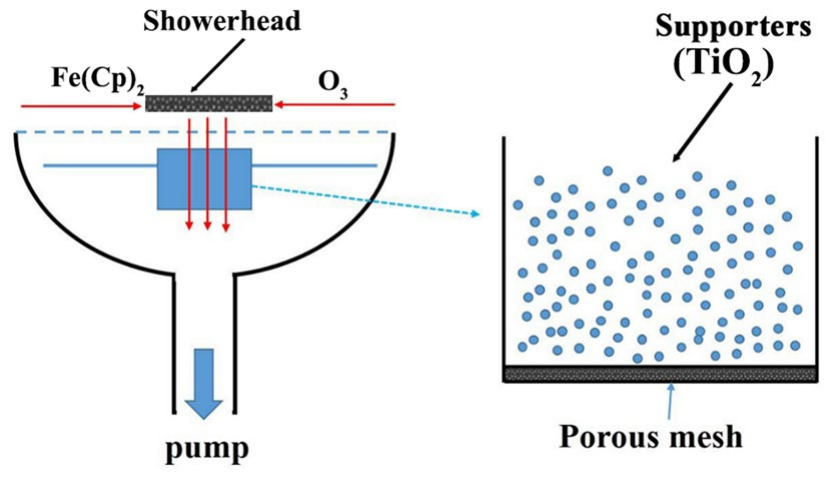
The schematic diagram of coating TiO_2_ powders by ALD Fe_2_O_3_.

Commercial TiO_2_ powders with anatase phase (Nanjing Haitai nano materials Co.) were used as supports in this work. Ferrocene (Fe(Cp)_2_, Suzhou Fornano Corporation Ltd., 99.99%) and ozone were adopted as Fe and oxygen precursors for ALD Fe_2_O_3_ deposition. Fe(Cp)_2_ was vaporized at 85 °C. High purity nitrogen gas (N_2_, 99.999%) was used as carrier gas at a total flow rate of 750 sccm and a pressure of 6 hPa in our ALD system (Picosun SUNALE™ R-150B). A particular container with porous mesh was used for ALD coating on powders, which has been reported elsewhere^24,55^, as shown in Fig. 1. Herein, precursors can flow through the TiO_2_ powders to achieve great conformality. X cycles of ALD Fe_2_O_3_ (X = 200, 400, 600, and 800) were coated on TiO_2_ powder at 300 °C, the samples are marked as TiO_2_@X-Fe_2_O_3_. One cycle of ALD Fe_2_O_3_ contains the following four steps, 5 s Fe(Cp)_2_ injection, 20 s N_2_ purge, 5 s O_3_ injection, and 20 s N_2_ purge. At the same system, 20 cycles of Al_2_O_3_ were deposited on TiO_2_@400-Fe_2_O_3_ at 300 °C, where one ALD cycle of Al_2_O_3_ is consisted of 5 s trimethylaluminum dose, 20 s N_2_ purging, 5 s H_2_O dose, and 20 s N_2_ purging.

### Materials characterizations

X-ray diffraction (XRD) using a Rigaku-D/MAX 2000 system was used for crystallinity and phase structure analysis. Scanning electron microscopy (SEM) images were taken using ZEISS Gemini SEM 500 instrument operated at 2 kV. The high-resolution transmission electron microscopy (HRTEM) was performed on a FEI Tecnai F20 S-Twin to observe the microstructures, where TiO_2_ powders were loaded on the ultra-thin carbon coated copper grids. The surface chemical features and valence band spectra were explored by X-ray photoelectron spectroscopy (XPS) using Thermo Fisher K-Alpha. The adventitious carbon signal (C 1 s = 284.6 eV) was adopted to calibrate the binding energies. UV–visible absorption spectra were conducted on a UV–vis-NIR spectrophotometer (UV-3600, Shimadzu, Japan). Photoluminescence (PL) spectra were collected on a Horiba Jobin Yvon HR800 spectrometer.

### Photocatalytic degradation

The photocatalytic performance of Fe_2_O_3_ coated TiO_2_ catalysts was investigated by visible light degradation of MO. 100 ml MO solution (4 mg L^−1^) with 100 mg photocatalysts were loaded into a glass reactor, which was magnetically stirred at 500 rpm. In order to establish the adsorption/desorption equilibrium between MO and catalysts before irradiation, the MO solutions with catalysts were magnetically stirred for 30 min in darkness. Then, the suspension was irradiated under a 300 W Xe lamp (MircoSolar300, Perfect Light). A 420 nm filter was adopted to cut off UV light. The lamp was placed at 15 cm above the suspension, whose average visible light intensity is around 80 mW cm^−2^. Water cooling was applied throughout the experiment to maintain the temperature at 25 ± 1 °C. 3 mL solution was collected after each 15 min irradiation. The photocatalysts were removed by centrifugal separation. The residual MO concentration was determined using the absorption at 464 nm by UV–Vis–NIR spectrophotometer. The recycled usage experiment was performed for three times to explore the long-term stability of photocatalysts, e.g. after each photocatalytic experiment, the photocatalysts powders were gathered and rinsed by ethanol and water, then baked for 12 h at 100 °C. At last, a new MO solution was used to evaluate the photocatalytic activity of collected photocatalysts.

### Photoelectrochemical measurements

Photoelectrochemical measurements were performed in a three-electrode electrochemical cell at room temperature using 1 M Na_2_SO_4_ as the electrolyte. The TiO_2_ or TiO_2_@400-Fe_2_O_3_ on FTO were used as the working electrode. A Pt wire and Ag/AgCl were used as the counter electrode and the reference electrode, respectively. Photocurrent densities were collected by an electrochemistry workstation (CHI660E, Shanghai) using a potentiostatic method at 0.50 V. Light was chopped on and off cyclically. A solar simulator (300 W Xe lamp, MircoSolar300, Perfect Light) with a 420 nm cut-off filter provides the visible-light irradiation.

## Results

**Figure 2:**
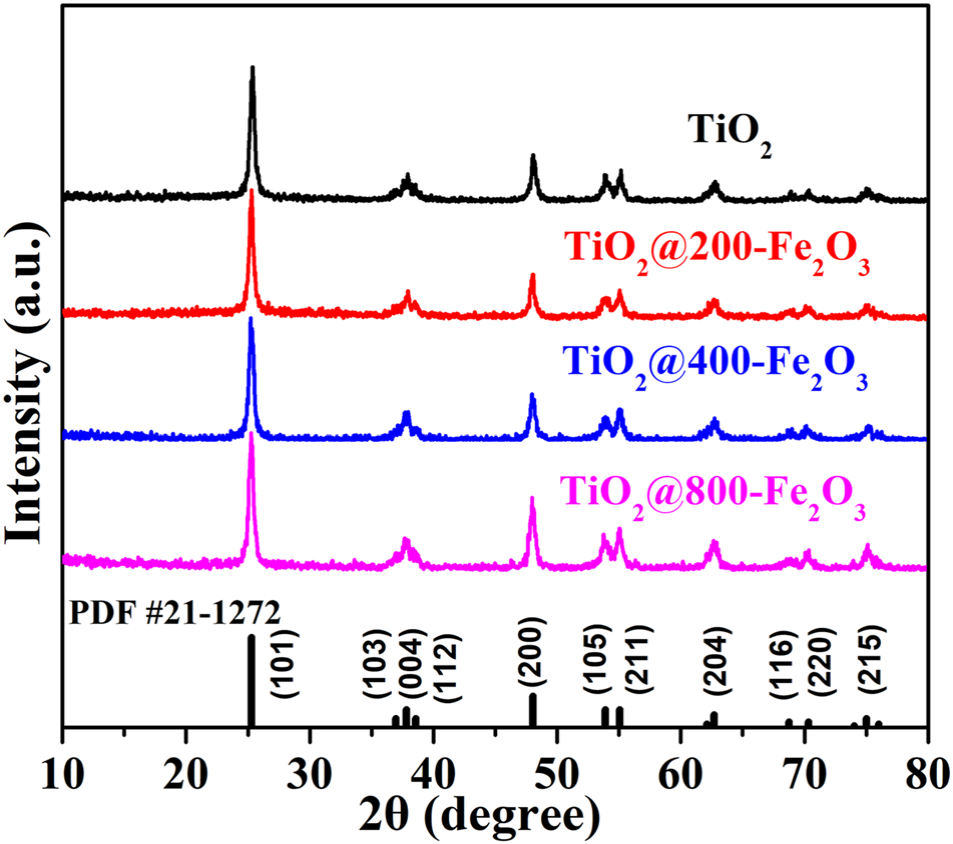
XRD patterns of pristine TiO_2_ and Fe_2_O_3_ coated TiO_2_.

Figure 2 depicts the XRD patterns of pristine TiO_2_ and Fe_2_O_3_ coated TiO_2_ powders. All the samples show the similar characteristic diffraction peaks, in accord with anatase TiO_2_ (JCPDS No. 21–1,272). This result indicates that ultrathin ALD Fe_2_O_3_ would not affect the crystal structure of TiO_2_, consistent with our previous finding^24,55^. In addition, signals related to Fe_2_O_3_ were absent.

**Figure 3:**
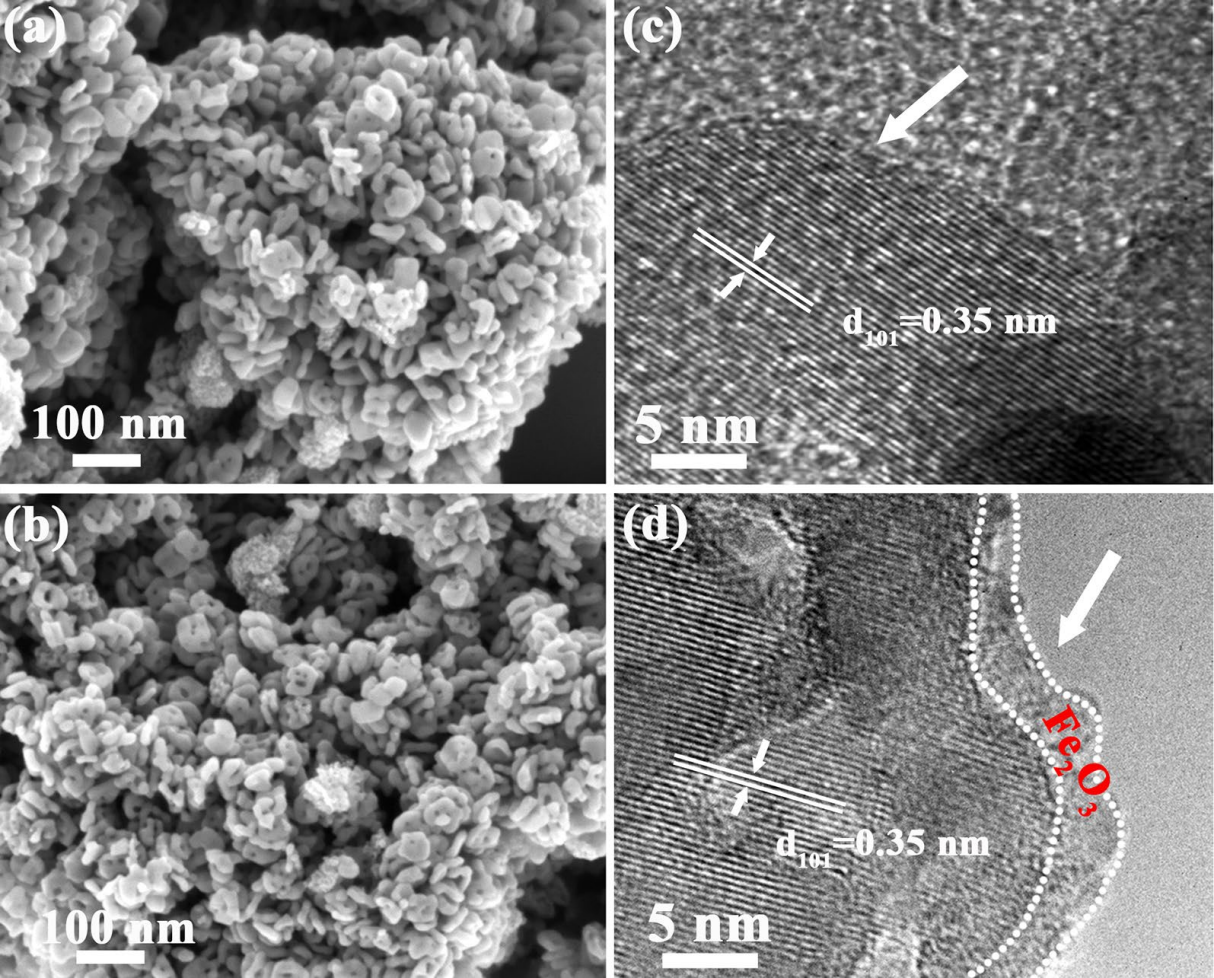
SEM images of (**a**) pristine TiO_2_ and (**b**) TiO_2_@400-Fe_2_O_3_. TEM images of (**c**) pristine TiO_2_ and (**d**) TiO_2_@400-Fe_2_O_3_.

SEM images of TiO_2_ powders without and with 400 cycles ALD Fe_2_O_3_ deposition are shown in Fig. 3a,b. It can be seen that the pristine TiO_2_ powder exhibits a disk-like morphology with a diameter of approximately 40 nm and a thickness of approximately 10 nm, with severe aggregation. After ALD Fe_2_O_3_ deposition, it was observed that Fe_2_O_3_ coated TiO_2_ exhibited almost identical morphology, indicating that ultra-thin Fe_2_O_3_ coating did not have significant influence on particle size and morphology of TiO_2_. HRTEM was further conducted to observe the microstructure of TiO_2_@400-Fe_2_O_3_ and pristine TiO_2_ powders as shown in Fig. 3c,d. While pristine TiO_2_ shows a sharp well-ordered surface with good crystallinity (Fig. 3c), an amorphous layer of ~ 2.6 nm formed on the TiO_2_ surface was observed which was contributed to ultra-thin Fe_2_O_3_ layer (400 cycles) formed by ALD deposition. Moreover, both samples exhibit the lattice spacing of 0.35 nm, corresponding to (101) planes of anatase TiO_2_. Based on above XRD and TEM data, it was speculated that an ultra-thin amorphous Fe_2_O_3_ is coated on TiO_2_ nanoparticles surface without significantly modifying the morphology of the catalyst supports.

**Figure 4:**
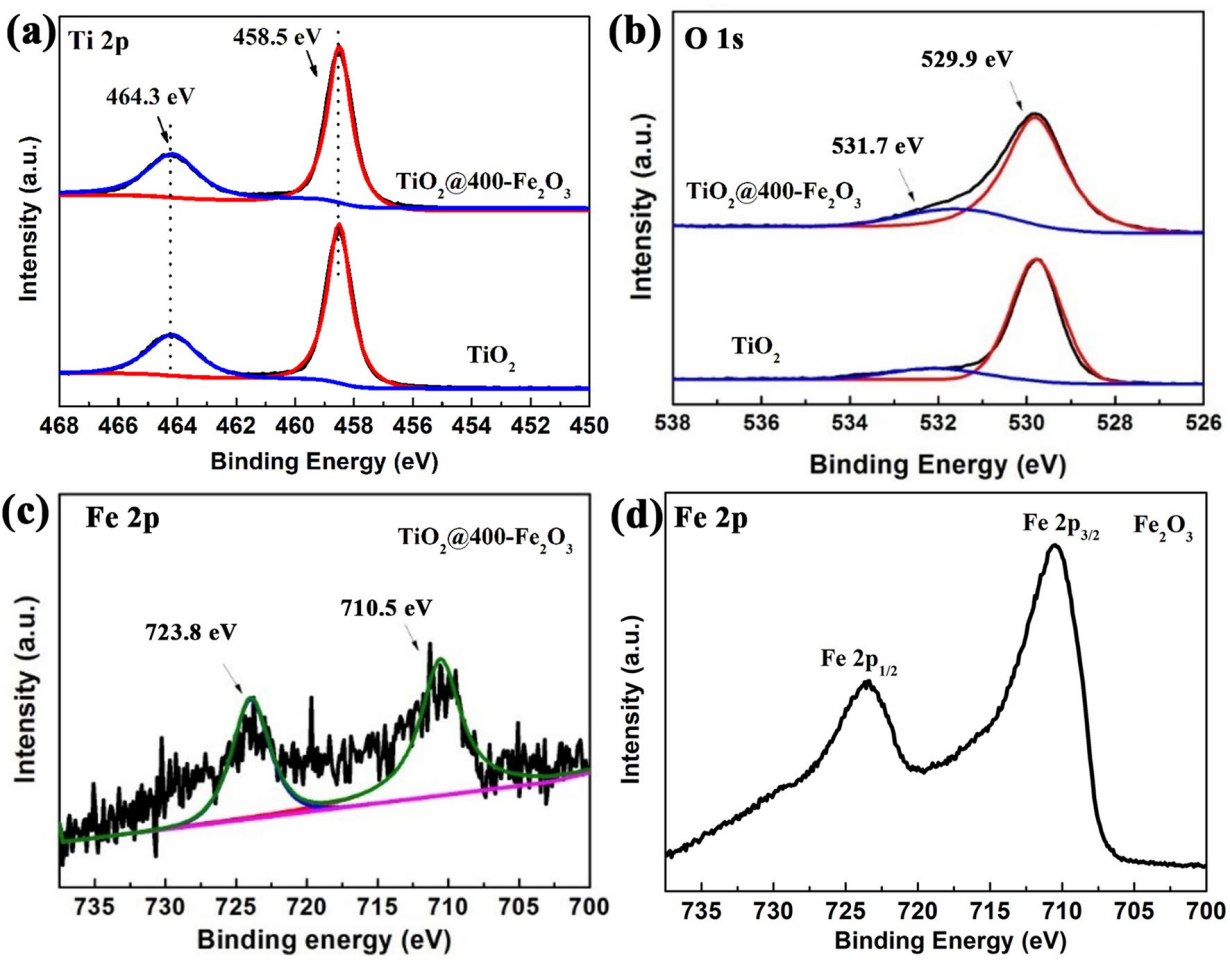
XPS spectra of (**a**) Ti 2p and (**b**) O 1s for TiO_2_ and TiO_2_@400-Fe_2_O_3_, Fe 2p XPS spectra for (**c**) TiO_2_@400-Fe_2_O_3_ and (**d**) Fe_2_O_3_ film.

To further determine the successful deposition of ALD Fe_2_O_3_, XPS was performed to characterize the surface chemical features of ALD Fe_2_O_3_ coated TiO_2_ powders. Figure 4a shows the Ti 2p spectra of TiO_2_@400-Fe_2_O_3_ and pristine TiO_2_ powders. The spectra can be fitted into two peaks at 464.3 eV and 458.5 eV, which can be assigned to Ti 2p_1/2_ and Ti 2p_3/2_ peaks^28^. In O 1 s spectra (Fig. 4b), both samples present the main peak at 529.9 eV related to Ti–O bonding from TiO_2_. In addition, there is a peak at 531.7 eV can be ascribed to the surface -OH^56^. Figure 4c exhibits the Fe 2p spectrum of TiO_2_@400-Fe_2_O_3_, Fe 2p_1/2_ and Fe 2p_3/2_ peaks locate at 723.8 eV and 710.5 eV, in accord with Fe–O bonding value in Fe_2_O_3_^57^. Based on XPS data, the Fe atom ratio (Fe/Fe + Ti) is determined to be 1.1% in TiO_2_@400-Fe_2_O_3_. It is anticipated that an ultra-thin amorphous Fe_2_O_3_ is coated on TiO_2_ nanoparticles surface successfully. Due to the low content of Fe_2_O_3_, Fe 2p spectrum shows a bad signal to noise ratio. Therefore, the Fe 2p spectrum of ALD Fe_2_O_3_ film is also presented for reference, as shown in Fig. 4d which can exhibit much better signal to noise ratio.

**Figure 5:**
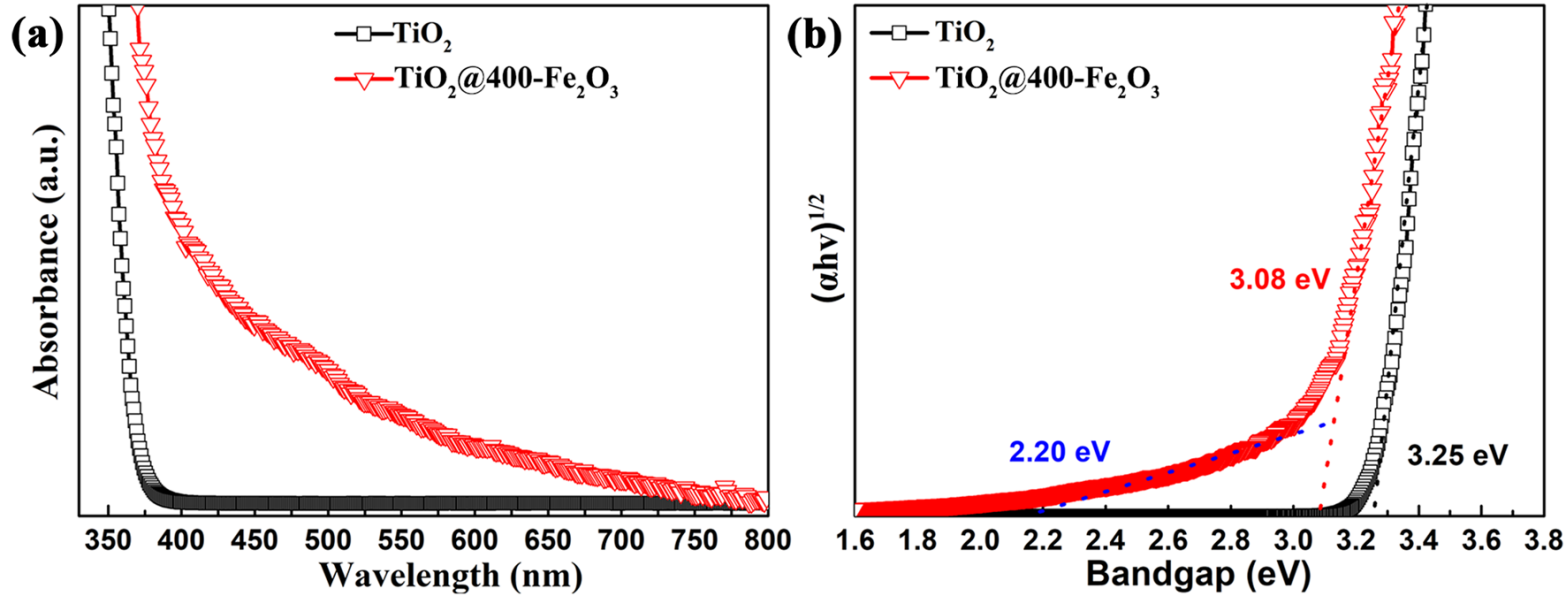
(**a**) UV–Vis diffuse reflectance spectra and (**b**) Tauc plot of TiO_2_ with and without 400 cycles Fe_2_O_3_ coating.

The influence of ultra-thin Fe_2_O_3_ coating on absorption of TiO_2_ powders in visible light was explored using UV–Vis diffuse reflectance spectra, as illustrated in Fig. 5a. The spectrum of pristine TiO_2_ powders is also plotted for comparison. Pristine TiO_2_ powders exhibit the absorption edge of around 371 nm without noticeable visible light absorption. However, noticeable absorption in the visible light region from 390 to 750 nm can be observed after ultra-thin Fe_2_O_3_ surface modification. The relationship of the absorption edge with the photon energy (*h*ν) for the indirect bandgap semiconductor is shown in the following formula: (αhν)^1/2^ = A(hν-E_g_), where α and A are the absorption coefficient and absorption constant, respectively. Since the absorption coefficient α is determined by the scattering and reflectance spectra based on Kubelka–Munk theory, therefore, the bandgap values can be determined by the intercept of the tangent lines. As depicted from Fig. 5b, only one tangent line can be extrapolated for pristine TiO_2_ powders showing a bandgap of 3.25 eV, while two bandgap values can be obtained from the plots for TiO_2_@400-Fe_2_O_3_ powders, attributing to Fe_2_O_3_ coating with a bandgap value of ~ 2.20 eV and TiO_2_ supports with a bandgap value of ~ 3.08 eV. It can be concluded that ultra-thin Fe_2_O_3_ coating results in a smaller bandgap which can increase the absorption of TiO_2_ powder support in visible light.

**Figure 6:**
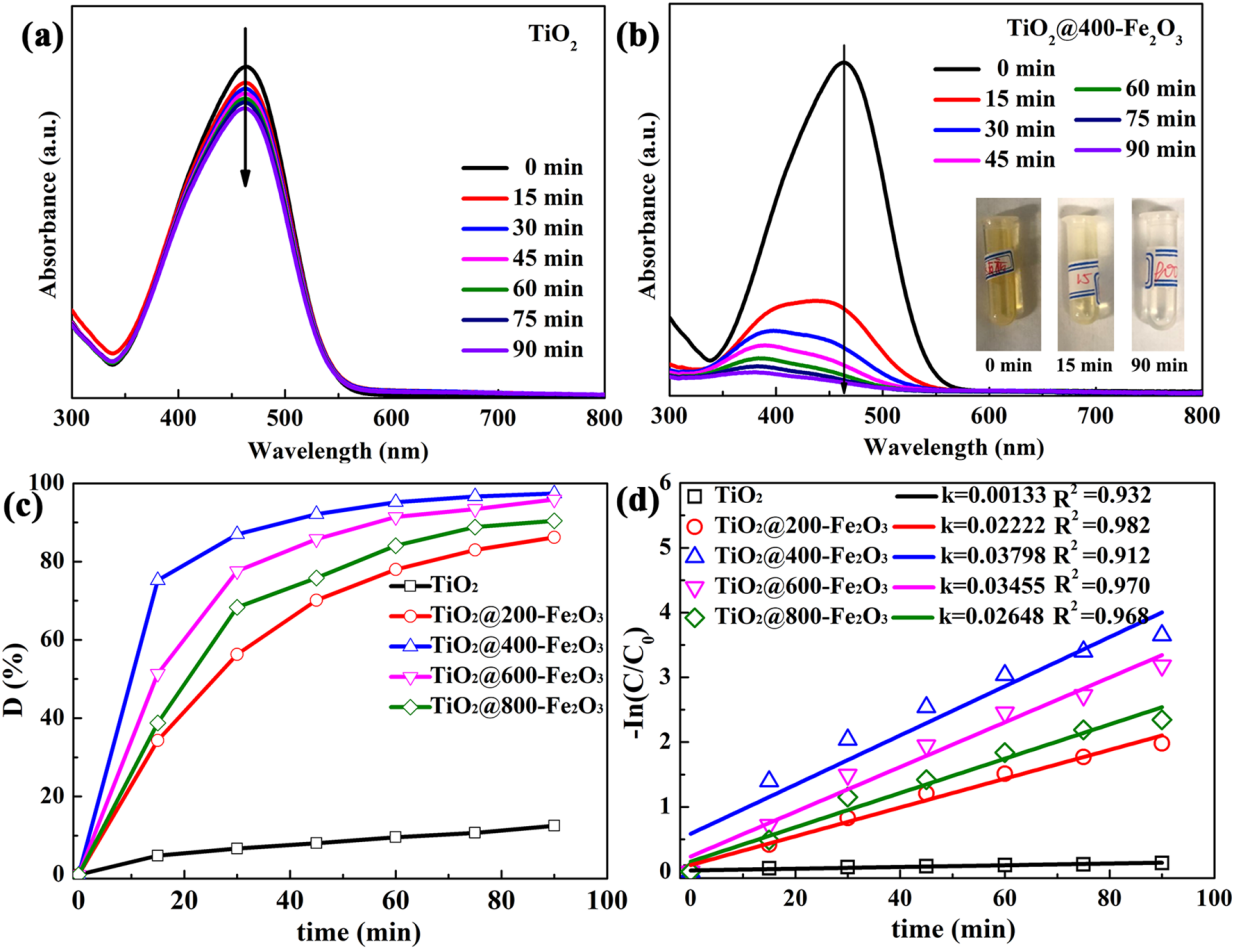
UV–vis absorption spectra of MO exposed to different irradiation time in the presence of (**a**) pristine TiO_2_ and (**b**) TiO_2_@400-Fe_2_O_3_ catalysts under visible light irradiation. The inserts are the photos of MO solution before and after irradiation. (**c**) Visible light photocatalytic degradation curves of MO and (**d**) −In(C/C_0_) vs. time curves by using TiO_2_ with and without Fe_2_O_3_ coating as catalysts.

The visible light photocatalytic activity of TiO_2_ and Fe_2_O_3_ coated TiO_2_ catalysts was compared by degrading MO. All samples exhibit low adsorption capacity of MO molecules. As reported in our previous work, MO is selected here for its stability under visible light irradiation in the absence of catalysts^24^. Figure 6a shows the evolution of UV–vis absorption spectra of MO solution in the presence of pristine TiO_2_ under visible light irradiation. It can been seen that the absorption peaks at 464 nm decreases slightly after 90 min, exhibiting very poor photocatalytic activity due to its large bandgap. In contrast, the peak intensity at 464 nm fades rapidly for TiO_2_@400-Fe_2_O_3_ with the irradiation time extending, as shown in Fig. 6b. In addition, the orange MO solution turns into colorless after 90 min, as presented in the insert of Fig. 6b, indicating the degradation of MO. Figure 6c compares visible light photocatalytic activity of Fe_2_O_3_ coated TiO_2_ catalysts. It can be found that a much-improved photocatalytic degradation efficiency of ~ 86.2% can be achieved with only 200 cycles of ALD Fe_2_O_3_ modification. And the TiO_2_@400-Fe_2_O_3_ photocatalysts display the highest photocatalytic degradation efficiency of 97.4%. In comparison with reported Fe_2_O_3_-TiO_2_ heterojunction catalysts for photodegradation of organic pollutants and antibiotics, ALD Fe_2_O_3_ coated TiO_2_ (TiO_2_@400-Fe_2_O_3_) in this work exhibit excellent removal efficiency for MO degradation, as summarized in Table 1. The photocatalytic degradation efficiency decreases to 95.8% and 90.4% for TiO_2_@600-Fe_2_O_3_ and TiO_2_@800-Fe_2_O_3_ samples, respectively, along with further increasing the thickness of ALD Fe_2_O_3_ coating. The reduced efficiency can be ascribed to the fact that more Fe_2_O_3_ coating would introduce more recombination sites for photoinduced electron–hole pairs^42^, diminishing the photocatalytic efficiency.

**Table 1:** Comparison of photocatalytic activity for degradation of organic pollutants using TiO_2_–Fe_2_O_3_ based catalysts.

Catalysts	Method	Power of Xe lamp (W)	Organic pollutants		Time (min)	D (%)	Ref
			type	C (mg L^-1^)			
Fe_2_O_3_ decorated TiO_2_	calcination	350	MB^a^	3.2	80	64.5	^58^
Fe_2_O_3_-Doped TiO_2_	Sol–gel	500	MB	10	120	100	^44^
Fe_2_O_3_/TiO_2_ nanofibers	Electrospinning + calcination	800	RhB^b^	5	180	53.6	^59^
Fe_2_O_3_ decorated TiO_2_	hydrothermal	500	RhB	4.8	270	77.8	^45^
Fe_2_O_3_@SiO_2_@TiO_2_	vapor-thermal	300	RhB	4.8	60	100	^46^
Fe_2_O_3_ coated TiO_2_	solvothermal	300	TC^c^	50	90	100	^39^
Core–shell Fe_2_O_3_@TiO_2_	Precipitation	350	RhB	10	360	71.0	^60^
Core–shell TiO_2_@Fe_2_O_3_	hydrothermal	300	MO	10	16	96.6	^43^
Core–shell C@TiO_2_@Fe_2_O_3_	Impregnation	500	MB	20	240	80.8	^61^
Fe_2_O_3_/TiO_2_ composites	Impregnation	500	Orange II	20	240	53.4	^62^
Fe_2_O_3_ coated TiO_2_	ALD	300	MO	4	90	97.4	This work

^a^ MB is Methylene Blue, ^b^ RhB is Rhodamine B, ^c^ TC is Tetracycline.

The degradation data were also fitted by the pseudo-first-order kinetics. The rate constant k can be determined by In(C_t_/C_0_) = −kt at low initial pollutant concentration. Herein, C_0_ is the initial MO concentration, while the C_t_ is the MO concentration after irradiation time of t. k is the first-order rate constant (min^−1^). The −In(C_t_/C_0_) vs. t curves are plotted in Fig. 6d. It can be seen that −In(C_t_/C_0_) has a linear relationship with t, indicating the photocatalytic degradation of MO by Fe_2_O_3_ modified TiO_2_ catalysts obeys the first-order kinetics. The first-order rate constant (k) is determined to be 3.8 × 10^−2^ min^−1^ for TiO_2_@400-Fe_2_O_3_, which is much larger than pristine TiO_2_ of 1.3 × 10^–3^ min^−1^. The result indicates that ALD Fe_2_O_3_ modification can effectively enhance the visible light photocatalytic performance of TiO_2_ supports.

**Figure 7:**
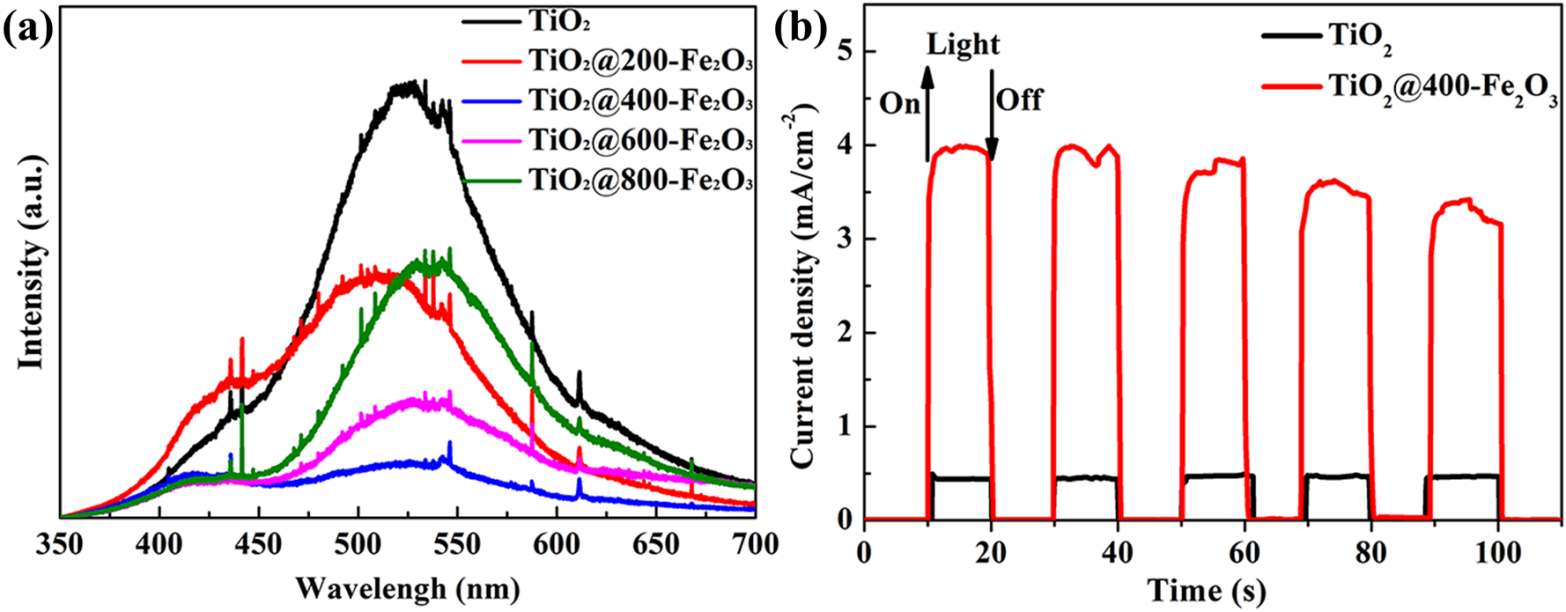
(**a**) PL spectra and (**b**) photocurrent response curves of TiO_2_ and Fe_2_O_3_ coated TiO_2_.

PL and photocurrent response measurements were conducted to explore the recombination rate of photo-generated electron–hole pairs. The lower the PL intensity, the higher separation efficiency of electron–hole pairs in the catalysts. Figure 7a shows the PL spectra of pristine TiO_2_ and various cycles of ALD Fe_2_O_3_ coated TiO_2_. It can be seen that all the Fe_2_O_3_ coated TiO_2_ exhibit lower intensity than pristine TiO_2_, indicating that the coupling of TiO_2_ and Fe_2_O_3_ can effectively inhibit the recombination of the photo-generated electron–hole pairs. Moreover, it can be seen that the 400 cycles Fe_2_O_3_ coating results in the lowest intensity, thicker Fe_2_O_3_ coating would increase the recombination of the photo-generated electron–hole pairs. The PL data are consistent well with the results of photocatalytic degradation of MO. Figure 7b presents the photocurrent response curves of TiO_2_ and TiO_2_@400-Fe_2_O_3_, it can be seen that TiO_2_@400-Fe_2_O_3_ exhibits a much higher photocurrent density than pristine TiO_2_, indicating a more efficient separation of the photoexcited electron–hole pairs.

**Figure 8:**
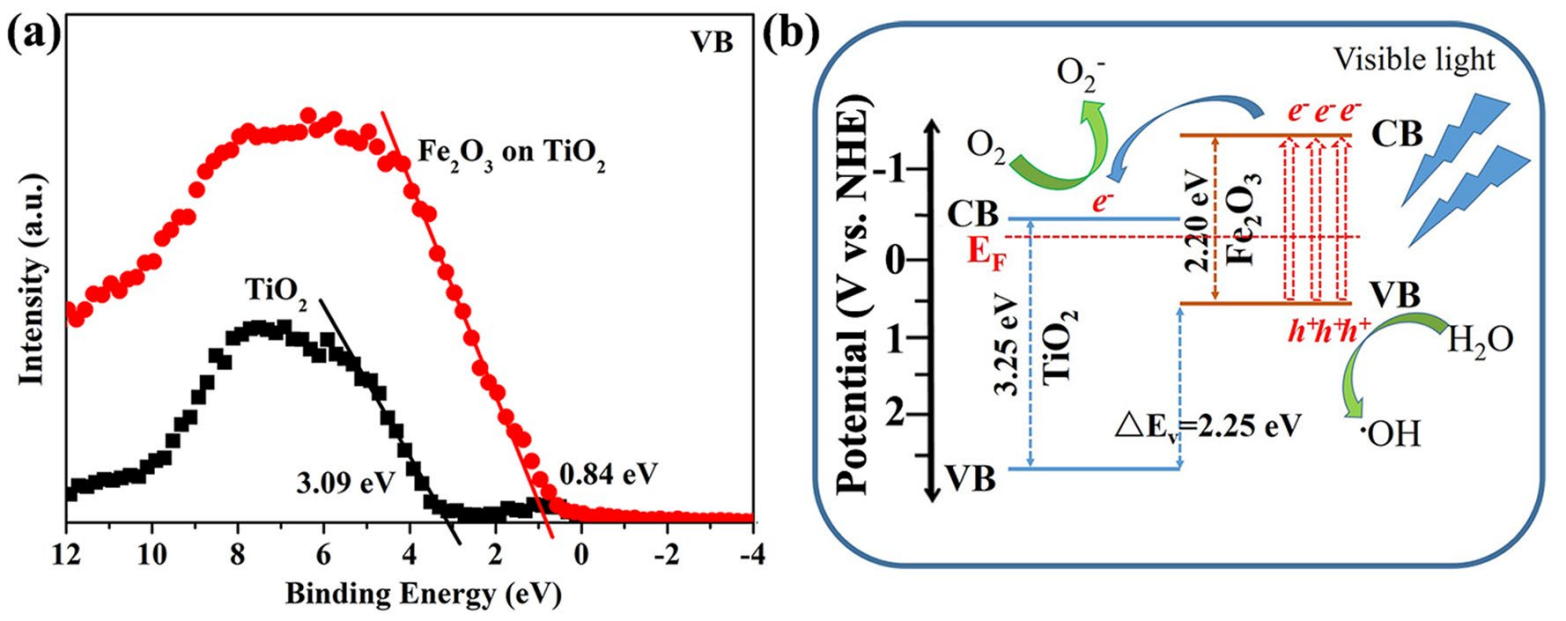
(**a**) Valence band spectra of TiO_2_ and Fe_2_O_3_–TiO_2_. (**b**) Schematic illustration of energy band structure of Fe_2_O_3_ coated TiO_2_ and proposed charge transfer mechanism during visible light irradiation.

The band alignment for Fe_2_O_3_ coated TiO_2_ was determined by measuring the valence band offset ∆E_*v*_ (VBO) using XPS. Figure 8a shows VB spectra of TiO_2_ and Fe_2_O_3_–TiO_2_ determined by linear extrapolation method, respectively. The VB of TiO_2_ and Fe_2_O_3_–TiO_2_ are found to be 3.09 eV and 0.84 eV, respectively. The VB of Fe_2_O_3_ is higher than that of TiO_2_, and ∆E_*v*_ at the interface of Fe_2_O_3_-TiO_2_ is estimated to be 2.25 eV. The optical bandgaps of TiO_2_ and Fe_2_O_3_ have been determined to be 3.25 and 2.20 eV, respectively, in Fig. 5. Therefore, the conduction band offset ∆E_*c*_ (CBO) at the interface of Fe_2_O_3_-TiO_2_ is estimated to be 1.20 eV. Considering the band structure of TiO_2_ vs. standard hydrogen electrode (NHE)^62–64^, the energy band structure of Fe_2_O_3_ coated TiO_2_ can be depicted in Fig. 8b. Under visible light, TiO_2_ shows no photo-electronic response due to its large band gap. Only Fe_2_O_3_ can be excited, yielding photo-generated electron from its VB to CB. Due to the aligned equilibrium of Fermi level at the interface of TiO_2_ and Fe_2_O_3_, as shown in Fig. 8b, the photogenerated electrons can transfer from CB of Fe_2_O_3_ to that of TiO_2_ driven by the built-in electric field and the concentration gradient of electrons, while remaining the holes in VB of Fe_2_O_3_^60,62–64^. Therefore, the separation efficiency of photoinduced electron–hole pairs can be improved, which has been demonstrated by PL and photocurrent response results in Fig. 7. There are a large number of literatures focusing on the photocatalytic activity of Fe_2_O_3_-TiO_2_ composites catalyst^60,62–64^. It is widely accepted that OH^**.**^ radicals can be formed via the reaction of water and photogenerated holes in VB of Fe_2_O_3_. And electrons in CB of TiO_2_ can react with oxygen to form O_2_^-^. These radicals with high activities can degrade organic molecules into harmless substances.

**Figure 9:**
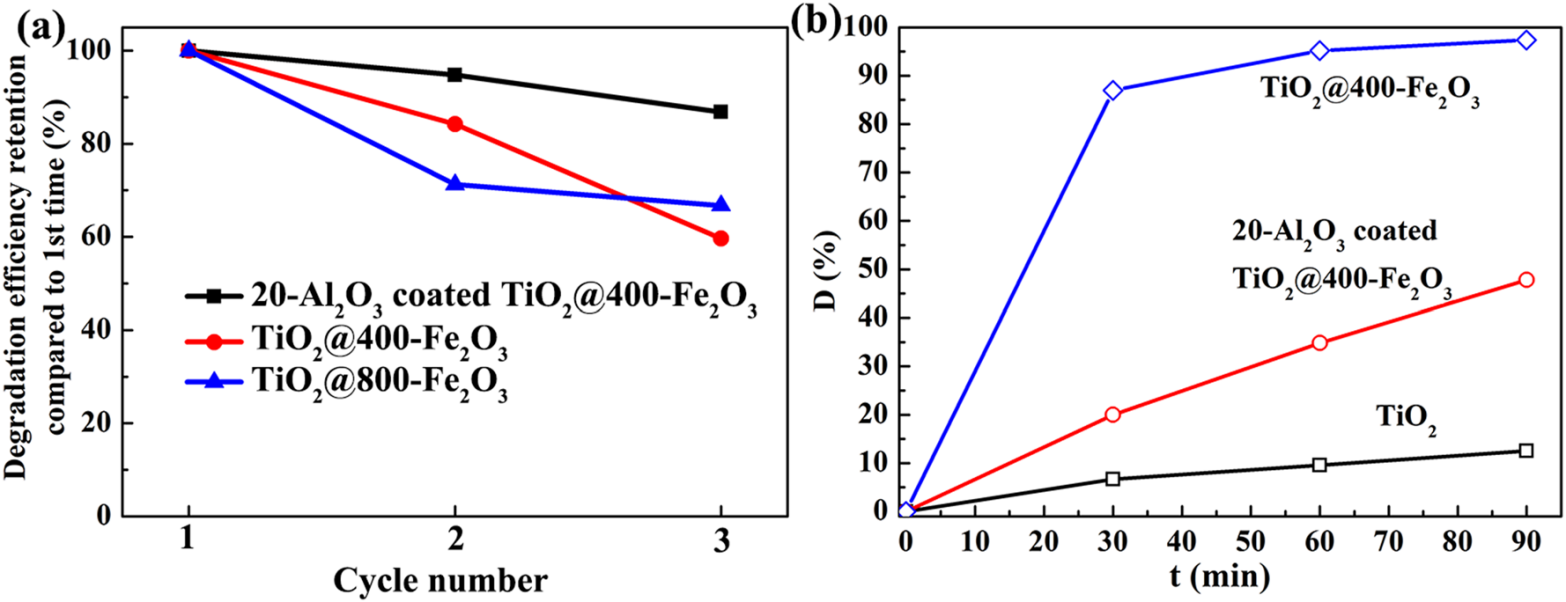
(**a**) Three cycles of MO degradation for TiO_2_@400-Fe_2_O_3_, TiO_2_@800-Fe_2_O_3_ and 20 cycles Al_2_O_3_ coated TiO_2_@400-Fe_2_O_3_ in 90 min. (**b**) Comparison of MO degradation curves for pristine TiO_2_, TiO_2_@400-Fe_2_O_3_, and 20 cycles Al_2_O_3_ coated TiO_2_@400-Fe_2_O_3_.

The stability of photocatalysts is one of the significant factors for practical applications, therefore, TiO_2_@400-Fe_2_O_3_ and TiO_2_@800-Fe_2_O_3_ catalysts were tested in recycling experiments of MO photodegradation. As shown in Fig. 9a, compared to the first usage, both TiO_2_@400-Fe_2_O_3_ and TiO_2_@800-Fe_2_O_3_ catalysts exhibit a declining degradation efficiency. The degradation efficiency retention compared to the first usage is only 59.6% and 66.7% for TiO_2_@400-Fe_2_O_3_ and TiO_2_@800-Fe_2_O_3_, respectively. It may be ascribed to the fact that stability of Fe_2_O_3_ during photocatalytic reactions is reduced due to photo-corrosion^42,65^. ALD coatings have been widely used as surface protection layer to protect active materials from photo-corrosion^53,54^. Therefore, an ultrathin Al_2_O_3_ (~ 2 nm) protective layer was deposited on catalysts surface by ALD in the same system to improve the stability of Fe_2_O_3_ coated TiO_2_ catalysts. Figure 9b shows the photocatalytic degradation of MO using both TiO_2_@400-Fe_2_O_3_ with Al_2_O_3_ passivation. The photocatalytic degradation efficiency decreases to 47.8% in 90 min, which is ascribed to the fact that Al_2_O_3_ coating with large band gap would also hinder the photogenerated carriers form migrating to the surface of electrode^62,66^. But it is still much better than that of pristine TiO_2_. More importantly, the stability of catalysts is persistent, as there is limited decline of degradation efficiency after three usage as shown in Fig. 8a, retaining 86.8% compared to the first usage. The results indicate that thin Al_2_O_3_ can act as a physical shell to protect Fe_2_O_3_ from photo-corrosion. This can be ascribed to that Al_2_O_3_ can prevent the direct contact between solution and Fe_2_O_3_.

### Conclusions

In summary, commercial anatase TiO_2_ powders were modified using ultrathin ALD Fe_2_O_3_ surface coating. The ultrathin Fe_2_O_3_ layer with small bandgap of ~ 2.20 V can increase the absorption of TiO_2_ supports in visible light. In addition, Fe_2_O_3_/TiO_2_ heterojunction can suppress the photoinduced electron–hole pairs recombination. The above results indicate excellent visible light photocatalytic activity for the Fe_2_O_3_ modified TiO_2_ powders. 400 cycles Fe_2_O_3_ (~ 2.6 nm) coated TiO_2_ photocatalysts show excellent degradation efficiency of 97.4% in 90 min, far above the performance of pristine TiO_2_ powders with only 12.5%. Moreover, an ultrathin Al_2_O_3_ (~ 2 nm) can improve the recycling usage performance of Fe_2_O_3_ coated TiO_2_ catalyst effectively. As conclusions, ALD surface modification with ultrathin film is a promising route for improving the visible light activity and long-term stability of photocatalysts.
